# Strangulated Amyand's hernia with testicular necrosis in an adult: A case report

**DOI:** 10.1016/j.ijscr.2025.110856

**Published:** 2025-01-06

**Authors:** Ephrem Adane Andargie, Suleiman Ayalew Belay, Michael A. Negussie, Hiwot Tesfaselassie Afework, Melaku Tessema Kassie, Hewan Fiseha Gebresellassie

**Affiliations:** a Department of Medicine, College of Medicine and Health Sciences, University of Gondar, Gondar, Ethiopia; b School of Medicine, College of Medicine and Health Sciences, University of Gondar, Gondar, Ethiopia; c School of Medicine, College of Health Sciences, Addis Ababa University, Addis Ababa, Ethiopia; d Department of Surgery, College of Medicine and Health Sciences, University of Gondar, Gondar, Ethiopia

**Keywords:** Amyand's hernia, Appendix, Strangulated, Testicular necrosis, Case report

## Abstract

**Introduction:**

Amyand's hernia is a rare condition defined by the presence of the vermiform appendix within an inguinal hernia sac. The occurrence of Amyand's hernia with testicular necrosis is particularly uncommon, further complicating its clinical presentation and management.

**Case presentation:**

A 50-year-old male presented with a two-year history of progressive right scrotal swelling, acutely worsened over four days with pain and fever. Examination revealed a firm, tender, irreducible right scrotal mass with overlying erythema. Laboratory tests showed leukocytosis. Imaging confirmed an inflamed appendix within the hernial sac, consistent with Amyand's hernia complicated by abscess formation. Emergency surgery revealed a gangrenous appendix, scrotal abscess, and necrotic right testicular tissue. The patient underwent appendectomy, orchiectomy, hernia repair, and abscess drainage. He recovered uneventfully, with symptom resolution and no recurrence at follow-up.

**Discussion:**

The progression of Amyand's hernia to appendicitis and subsequent perforation, as seen in our case, can result in severe complications, including abscess formation and testicular necrosis. The Losanoff and Basson classification categorizes Amyand's hernia based on the appendix's condition and associated complications, ranging from a normal appendix (Type 1) to severe extra-sac pathology such as gangrene or malignancy (Type 4). Our case aligns with Type 4, involving a perforated appendix with gangrene and a scrotal abscess, necessitating extensive surgical intervention.

**Conclusion:**

This case highlights the rarity and complexity of Amyand's hernia in adults, emphasizing the need for prompt recognition and tailored management to achieve favorable outcomes.

## Highlights

• Strangulated Amyand's hernia with testicular necrosis is rare in adults.
• The patient presented with acute scrotal swelling, fever, and erythema; imaging revealed a gangrenous appendix.
• Emergency surgery included appendectomy, orchiectomy, hernia repair, and abscess drainage.
• Postoperative recovery was smooth, with no recurrence or complications at follow-up.

## 1 Introduction

First described in 1735 by Claudius Amyand, Amyand's hernia refers to the presence of the vermiform appendix, whether normal or inflamed, within an inguinal hernial sac [1,2]. Recent studies indicate that Amyand's hernia has a prevalence of 0.4–0.6 % globally. However, in children, it is approximately three times more common, likely due to the presence of a patent processus vaginalis. [3]. Here we present a case of strangulated right-sided Amyand's hernia with testicular necrosis in an adult.

This case has been reported in line with the SCARE criteria [4].

## 2 Case presentation

A 50-year-old male presented with a two-year history of gradually progressive scrotal swelling, which had significantly worsened over the past four days. He also reported new-onset scrotal pain, low-grade fever, and increased swelling during this period. He denied any associated gastrointestinal symptoms such as nausea, vomiting, or changes in bowel habits. There was no history of prior hernia repairs, abdominal surgeries, or significant chronic medical conditions. The patient had no known drug allergies and was not on any regular medications.

Upon clinical examination, the patient appeared moderately distressed due to pain. He was tachycardic, with a pulse rate of 112 beats per minute, and febrile, with a recorded temperature of 38.4 °C. Blood pressure was 130/80 mmHg, and oxygen saturation was 98 % on room air. The abdomen was soft and non-tender, with no palpable masses or evidence of peritoneal irritation. Examination of the scrotum revealed a firm, irreducible, and tender mass approximately 4 × 5 cm in size over the right scrotum, accompanied by overlying erythema and warmth. The left testis and scrotal sac appeared normal. A digital rectal examination was performed and found to be unremarkable.

Laboratory investigations revealed a markedly elevated white blood cell (WBC) count of 21,300/mm^3^, indicative of an ongoing infection or inflammatory process. Serum electrolytes and renal function tests were within normal limits. Urinalysis showed no signs of urinary tract infection or hematuria.

A scrotal ultrasound identified a hernia within the right scrotal sac containing edematous mesentery and the inflamed tip of the appendix, along with a 3 cm fluid collection suggestive of an abscess. The left scrotum was normal. Abdominal ultrasound showed no abnormalities, ruling out other intra-abdominal sources of infection or pathology. Based on the clinical and imaging findings, a provisional diagnosis of a strangulated right-sided Amyand's hernia was made.

**Fig. 1:**
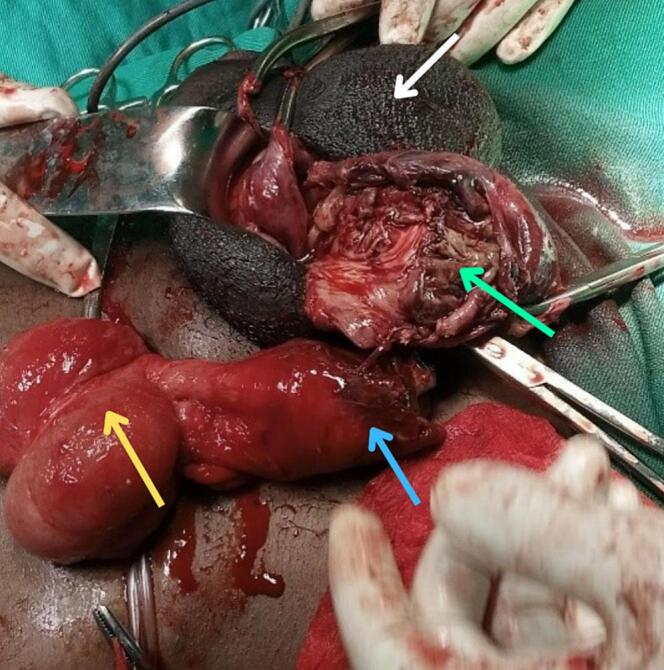
The white arrow indicates the scrotum. The green arrow shows necrotic tissue within the scrotum. The blue arrow points to the tip of the appendix. The yellow arrow points to the cecum. (For interpretation of the references to colour in this figure legend, the reader is referred to the web version of this article.)

**Fig. 2:**
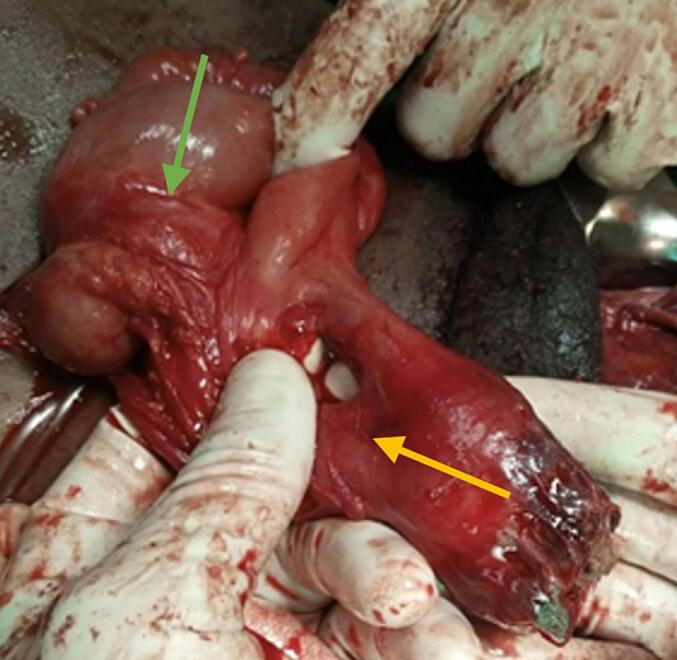
The yellow arrow indicates the mesoappendix, while the green arrow points to the cecum. (For interpretation of the references to colour in this figure legend, the reader is referred to the web version of this article.)

**Fig. 3:**
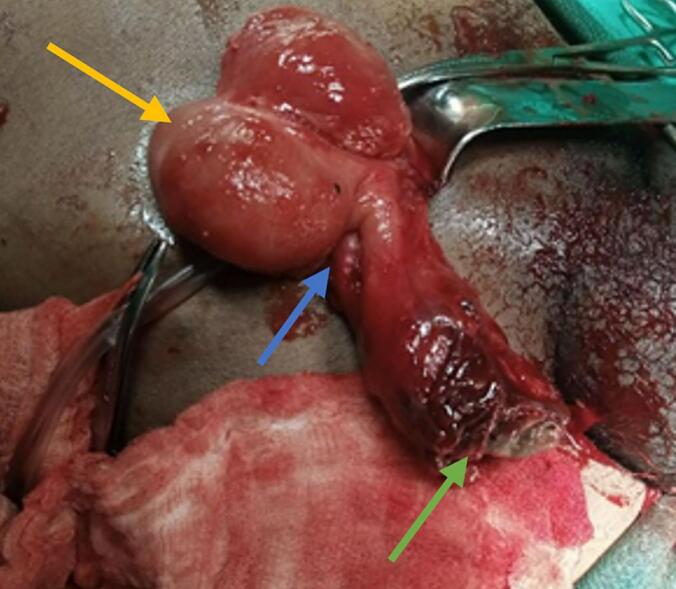
The yellow arrow represents the cecum, the blue arrow indicates the base of the appendix, and the green arrow marks the tip of the appendix. (For interpretation of the references to colour in this figure legend, the reader is referred to the web version of this article.)

The patient underwent urgent inguinal exploration under general anesthesia. A right oblique inguinal incision was made, revealing a hernial sac containing a gangrenous appendix that had descended into the scrotum. Approximately 70 mL of thick, foul-smelling pus was drained from the right scrotum, and extensive necrotic inflammatory tissue involving the distal appendix, hernial sac, distal spermatic cord, and the right testis was noted. These findings confirmed the diagnosis of a strangulated right-sided Amyand's hernia with associated abscess formation. The testicular tissue was grossly necrotic, and debridement was performed (Fig. 1). The mesoappendix was ligated and removed (Fig. 2, Fig. 3), followed by the identification and ligation of the spermatic cord and appendectomy. The necrotic testicular tissue was also excised. Modified Bassini repair was performed via an oblique inguinal incision after the herniated contents were reduced. The sac was ligated at the deep inguinal ring. The repair involved division of the external oblique aponeurosis, opening of the inguinal canal, and isolation of the spermatic cord. The conjoined tendon was sutured above with the inguinal ligament in a continuous fashion, from the pubic tubercle to the deep inguinal ring. The external oblique aponeurosis was then closed.

A separate transverse incision was made on the right scrotum for abscess drainage and additional debridement. Thorough irrigation with saline was performed, and a drain was placed to prevent fluid accumulation. Pathology was performed on the excised testicular tissue, which confirmed the presence of necrosis.

The patient's recovery was uneventful. He remained afebrile and hemodynamically stable following surgery. The patient was administered Ceftriaxone 1 g IV twice daily and Metronidazole 500 mg IV three times daily for the first 24 h postoperatively. On the following day, the patient was switched to oral antibiotics as enteral feeding was initiated. The drain was removed on the third postoperative day after minimal output was observed. He was discharged on the fourth postoperative day with a prescription for oral antibiotics and analgesics, and advised to maintain scrotal support and follow wound care instructions.

At his two-week follow-up, the patient reported significant improvement, with no recurrence of pain or swelling. Physical examination revealed well-healed surgical incisions, no signs of infection, and no evidence of recurrent hernia or fluid collection in the scrotal sac. A follow-up scrotal ultrasound confirmed the absence of any residual abscess or hernia.

## 3 Discussion

Amyand's hernia is most frequently reported in men, and almost exclusively on the right side. There is, however, an exception where the appendix is on the left side: situs inversus, intestinal malrotation, a very loose cecum or a large appendix [3,5,6].

The pathophysiology remains unsettled. One widely accepted explanation is that appendicitis may develop due to external compression from muscle contractions and sudden increases in intra-abdominal pressure, causing ischemia and subsequent inflammation [3]. Other mechanisms that can lead to acute appendicitis include adhesions causing irreducibility of the hernia and compression of the appendix in the external ring from increased intra-abdominal pressure [7]. In a few cases, Amyand's appendicitis has been associated with fecaliths or villous adenomas [8].

Patients with Amyand's hernia may present with symptoms resembling those of an incarcerated or strangulated inguinal hernia, including groin swelling, pain, and signs of systemic infection [9]. In this case, the patient exhibited a two-year history of progressive scrotal swelling, acutely exacerbated over four days, accompanied by scrotal pain, fever, and erythema. The absence of gastrointestinal symptoms such as nausea, vomiting, or changes in bowel habits is noteworthy, as it may obscure the underlying appendiceal involvement [10].

The progression of an Amyand's hernia to appendicitis can lead to severe complications, including abscess formation, necrosis of adjacent structures, and sepsis [11]. In this patient, the gangrenous appendix within the hernial sac resulted in a scrotal abscess and necrosis of the right testis, necessitating extensive surgical debridement. Such complications highlight the importance of prompt recognition and intervention to prevent morbidity [12].

The Losanoff and Basson classification of Amyand hernias defines four types based on the condition of the appendix and associated pathology. Type 1 involves a normal appendix within the hernia sac, typically managed with reduction or appendectomy and mesh hernioplasty if there are no inflammatory changes. Type 2 features acute appendicitis confined to the hernia sac, requiring appendectomy through the hernia and endogenous tissue repair due to the risk of mesh infection. Type 3 includes acute appendicitis with sepsis extending beyond the sac, such as peritonitis or necrotizing fasciitis, requiring appendectomy via laparotomy and additional procedures as needed, with hernioplasty deferred in critically ill patients. Type 4 involves hernias with acute appendicitis and coexisting serious extra-sac pathology, which may include conditions such as appendiceal cancer, or diverticulitis, necessitating appendectomy, diagnostic evaluation, and individualized surgical management based on the associated pathology and patient condition [13].

Our case is classified as a Type 4 Amyand's hernia because it involves acute appendicitis within the hernia sac accompanied by serious extra-sac pathology. The presence of a gangrenous appendix, a large scrotal abscess, and extensive necrotic inflammatory tissue involving the distal spermatic cord and testis clearly extends beyond the confines of the hernia sac.

Manatakis et al. conducted a 20-year systematic review of Amyand's hernia, which involved 231 studies describing 442 patients. The study reported that type 4 hernias, which include cases of intestinal, omental, or testicular necrosis, as well as cecal adenocarcinoma, represented a small portion of the overall cases, specifically around 2 % [14].

The surgical approach to Amyand's hernia depends on the condition of the appendix and the presence of infection [8]. In cases with a non-inflamed appendix, reduction and mesh hernioplasty are typically performed [15]. In the presence of appendicitis, as observed in our case, an appendectomy is necessary. The use of mesh is typically avoided due to the associated risk of infection [16]. Moreover, mesh was unavailable, and its high cost rendered it an impractical option for this case. A two-stage repair was also not considered, as the local tissue surrounding the inguinal incision was deemed adequate to hold sutures effectively. Similarly, Juan et al. managed a case of Amyand's hernia complicated by a perforated appendix and a scrotal abscess through an emergency appendectomy and herniorrhaphy using a modified Bassini method, without the use of prosthetic mesh [19]. The decision to perform orchiectomy in our case was based on the intraoperative finding of testicular necrosis, a rare but serious complication [17].

The prognosis for patients with Amyand's hernia largely depends on the timeliness of diagnosis and intervention [18]. Early surgical management can lead to favorable outcomes, as demonstrated in this case, where the patient experienced an uneventful recovery with no recurrence at follow-up. Delayed treatment, however, may result in increased morbidity due to complications such as abscess formation, bowel ischemia, or systemic infection [6].

## 4 Conclusion

This report adds to the understanding of Amyand's hernia by documenting a rare instance of appendiceal perforation with concurrent testicular necrosis. The need to consider atypical presentations and adapt treatment strategies accordingly is highlighted.

## Author contribution

**Ephrem Adane Andargie**: Writing – original draft, Conceptualization, Resources. **Suleiman Ayalew**: Writing – original draft, Conceptualization, Data curation. **Michael A. Negussie**: Writing – review & editing, Visualization. **Hiwot Tesfaselassie Afework**: Writing – review & editing, Data curation. **Melaku Tessema Kassie**: Writing – review & editing, Resources. **Hewan Fiseha Gebresellassie**: Supervision.

## Ethical approval

Ethical approval for this case report was provided by the School of Medicine, University of Gondar Ethics Board, Gondar, Ethiopia on November 11, 2024.

## Guarantor

Michael A. Negussie.

## Research registration number

N/A.

## Consent for publication

Written informed consent was obtained from the patient for publication of this case report and accompanying images. A copy of the written consent is available for review by the Editor-in-Chief of this journal on request.

## Funding

No source of funding is provided for this case report.

## Conflict of interest statement

The authors declare that they have no known competing financial interests or personal relationships that could have appeared to influence the work reported in this paper.
